# Probiotic supplementation during pregnancy or infancy for the prevention of asthma and wheeze: a systematic review and meta-analysis

**DOI:** 10.1186/1710-1492-10-S1-A25

**Published:** 2014-03-03

**Authors:** Meghan B Azad, J Gerard Coneys, Anita L Kozyrskyj, Catherine J Field, Clare D Ramsey, Allan B Becker, Carol Friesen, Ahmed M Abou-Setta, Ryan Zarychanski

**Affiliations:** 1 Pediatrics, University of Alberta, Edmonton, Alberta, Canada; 2 Department of Internal Medicine, University of Manitoba, Winnipeg, Manitoba, Canada; 3 Department of Community Health Sciences, University of Manitoba, Winnipeg, Manitoba, Canada; 4 Department of Agriculture, Food & Nutritional Sciences, University of Alberta, Edmonton, Alberta, Canada; 5 Manitoba Institute of Child Health, Winnipeg, Manitoba, Canada; 6 Pediatrics and Child Health, University of Manitoba, Winnipeg, Manitoba, Canada; 7 George & Fay Yee Centre for Healthcare Innovation, University of Manitoba / Winnipeg Regional Health Authority, Winnipeg, Canada; 8 Neil John Maclean Health Sciences Library, University of Manitoba Libraries, Winnipeg, Canada

## Background

Often preceded by early-life wheezing, asthma is the most common chronic disease of childhood. In view of the emerging microflora hypothesis of allergic disease, probiotics have been proposed for the prevention and treatment of allergic disorders including asthma, but clinical studies have yielded conflicting results. We undertook a systematic review and meta-analysis of randomized controlled trials to evaluate the association of probiotic use during pregnancy or infancy with childhood asthma and wheeze.

## Methods

Following a registered protocol (PROSPERO CRD42013004385), we searched MEDLINE, EMBASE, and CENTRAL from inception to February 2013, plus the World Health Organization's International Clinical Trials Registry Platform and relevant conference proceedings for the preceding 5 years. Included trials and relevant reviews were forward searched in Web of Science. Two reviewers independently identified randomized controlled trials evaluating probiotics administered during pregnancy or the first year of life. The primary outcome was clinician-diagnosed asthma; secondary outcomes included wheeze and lower respiratory tract infection.

## Results

We identified 20 eligible trials involving 4866 children. Studies were heterogeneous in the type and duration of probiotic supplementation, and duration of follow up. Two trials conducted follow-up at or beyond 6 years of age, and no trials were powered for asthma detection. We adjudicated most trials (16/20) to be of unclear or high risk of bias. Among 2781 infants enrolled in 8 studies contributing asthma data, the risk ratio of clinician-diagnosed asthma in participants randomized to receive probiotics was 0.96 (95% confidence interval [CI] 0.71 to 1.29, I^2^ = 13%). The risk ratio of incident wheeze was 0.95 (95%CI 0.85 to 1.07, I^2^ = 0%, 8 trials, 1770 infants). Among 1364 infants enrolled in 6 trials, the risk ratio of lower respiratory tract infection following probiotic use was 1.26 (95%CI 0.99 to 1.61, I^2^ = 0%).

## Conclusions

We found no evidence to support a protective association between perinatal administration of probiotics and clinician-diagnosed asthma or childhood wheeze. There is currently insufficient evidence from randomized controlled trials to recommend probiotics for the primary prevention of these disorders. Extended follow up of existing trials, along with further clinical and basic research, are required to accurately define the role of probiotics in the prevention of childhood asthma.

